# The effect of chronic kidney disease on CYP2B expression and activity in male Wistar rats

**DOI:** 10.1002/prp2.475

**Published:** 2019-04-26

**Authors:** Andrew S. Kucey, Thomas J. Velenosi, Nicholas C. Tonial, Alvin Tieu, Adrien A. E. RaoPeters, Brad L. Urquhart

**Affiliations:** ^1^ Department of Physiology and Pharmacology Schulich School of Medicine and Dentistry London Ontario Canada; ^2^ Lawson Health Research Institute London Ontario Canada; ^3^ Department of Medicine Division of Nephrology Schulich School of Medicine and Dentistry London Ontario Canada

**Keywords:** chronic kidney disease, cytochrome P450, hepatic drug metabolism, nonrenal clearance

## Abstract

Chronic kidney disease (CKD) is characterized by progressive reduction in kidney function over time. CKD affects greater than 10% of the population and its incidence is on the rise due to the growing prevalence of its risk factors. Previous studies demonstrated CKD alters nonrenal clearance of drugs in addition to reducing renal clearance. We assessed the function and expression of hepatic CYP2B enzymes using a rat model of CKD. CKD was induced in Wistar rats by supplementing their chow with adenine and confirmed through the detection of elevated uremic toxins in plasma. Liver enzymes AST and ALT were unchanged by the adenine diet. Bupropion was used as a probe substrate for hepatic CYP2B function using rat liver microsomes. The resulting metabolite, hydroxy‐bupropion, and bupropion were quantified by ultra‐performance liquid chromatography coupled to time‐of‐flight mass spectrometry. Level of mRNA and protein were determined by RT‐PCR and Western blot, respectively. The results of our study demonstrate that CYP2B1 is downregulated in a rat model of CKD. CYP2B1 mRNA level was significantly decreased (88%, *P *<* *0.001) in CKD relative to control. Similarly, maximal enzymatic velocity (*V*_max_) for CYP2B was decreased by 46% in CKD relative to control (*P *<* *0.0001). Previous studies involving patients with CKD demonstrated altered bupropion pharmacokinetics compared to control. Hence, our results suggest that these alterations may be mediated by attenuated CYP2B hepatic metabolism. This finding may partially explain the alterations in pharmacokinetics and nonrenal drug clearance frequently observed in patients with CKD.

**Abbreviations**
ALT: Alanine Aminotransferase
AST: Aspartate Aminotransferase
CKD: Chronic Kidney Disease
GFR: Glomerular Filtration Rate
*K*_m_: Michaelis‐Menten constant
QToF: Quadrupole Time of Flight
UPLC: Ultra Performance Liquid Chromatography
*V*_max_: Maximum rate of reaction of enzyme

## 1 INTRODUCTION

Chronic kidney disease (CKD) affects greater than 10% of the population and its prevalence has been on the rise due to an increased incidence of common risk factors such as diabetes mellitus and hypertension.^1^, ^23^, ^35^ CKD is defined as a progressive loss of renal function or significant pathological changes to renal structure which have been present for more than 3 months.^15^, ^26^, ^34^ A major clinical feature of this disease is the decline in glomerular filtration rate (GFR) due to kidney damage. Since the kidneys are a major site for xenobiotic processing and elimination, the impact of CKD extends beyond the primary function of the kidneys by dramatically altering drug clearance.^39^

Chronic kidney disease patients are prescribed a mean of 14.2 different drugs concurrently to manage their underlying condition as well as associated comorbidities such as diabetes, hypertension, and cardiovascular disease.^40^ This combination leads to an increased incidence of adverse drug events due to drug interactions and altered drug pharmacokinetics.^20^ Dosing alterations for some renally cleared drugs in CKD patients are fairly well characterized; however, nonrenal metabolism and transport have also been shown to be altered in CKD.^8^, ^24^, ^27^, ^28^, ^29^, ^37^, ^38^, ^45^ Explanations for this focus on alterations in transcriptional, translational, and nuclear regulation of hepatic or enteric drug metabolizing enzymes and transporters, which may be caused by increased levels of inflammatory cytokines and uremic toxins.^5^, ^17^, ^30^, ^44^, ^45^

The liver is the primary site for drug metabolism due mostly in part to the cytochrome P450 (CYP) superfamily of enzymes. Previous studies investigating changes of nonrenal clearance in CKD used animal models to demonstrate differences in function or expression of rat CYP1A2, CYP2C6, CYP2C11, CYP2D1/CYP2D2, CYP3A1, CYP3A2, CYP4A1/CYP4A3, as well as changes in the epigenetic regulation of CYP2C11 and CYP3A2.^9^, ^16^, ^18^, ^24^, ^32^, ^43^, ^44^, ^45^

This study focused on CYP2B isoenzymes since they have been minimally studied in the context of CKD despite having a clinically relevant role in the metabolism of many drugs, nutraceuticals, and herbal medicines. Specifically, the CYP2B6 enzyme alone is responsible for metabolism of 7.2% of prescribed medications (W.^33^, ^51^ Human CYP2B6 accounts for 6% of the total hepatic P450 content but has high interindividual and ethnic variation in expression. For example, 85% of Caucasians have detectable CYP2B expression in contrast to only 30% in a study from a Japanese population.^2^, ^21^, ^36^ CYP2B6 shares approximately 75% sequence homology with rat CYP2B1 and CYP2B2.^6^ Rat CYP2B1 and CYP2B2 are isoenzymes with 97% primary structural identity overlap and have similar substrate specificities.^48^ Catalytic activity is the primary differentiator between the two enzymes, with CYP2B1 being more active.^31^, ^48^ This is highlighted in a study that observed the metabolism of a CYP2B selective marker, bupropion (BUP), by rat liver microsomes. The paper reported that 75% of the BUP conversion to its metabolite OH‐bupropion (OH‐BUP) was attributable to CYP2B1, while CYP2E1 and CYP2C11 accounted for 10.9% and 8.7%, respectively.^31^

In humans 71% of the OH‐BUP formation is due to CYP2B6 and multiple studies have reported reductions in conversion of BUP to OH‐BUP in CKD patients.^4^, ^7^, ^12^, ^14^, ^42^ To date, no study has assessed the expression of CYP2B6 or the expression and function of rat CYP2B1 and CYP2B2 in CKD. Using a model of CKD induced by an adenine diet in male Wistar rats, we investigated the expression and function of CYP2B1 and CYP2B2.

## 2 MATERIALS AND METHODS

### 2.1 Rat model of CKD

The adenine model of CKD used was developed by adhering to a previously described protocol.^8^, ^46^ Male Wistar rats (150 g) were acquired from Charles River Laboratories and allowed a 5‐day acclimation period before commencement of the study diet. Animals were randomly divided into control and CKD groups (n = 9 and n = 12, respectively). Animals allocated to the CKD group were given a diet supplemented with 0.7% adenine for 5 weeks, followed by 3 weeks of control diet. Control animals were pair‐fed to the CKD group using standard rat chow for the entire study. Animals were euthanized using isoflurane as a general anesthetic, followed by subsequent decapitation at the conclusion of the study. Plasma and liver tissue were harvested from the animals. Samples were kept frozen at −80°C and −20°C for liver and plasma, respectively, until analysis. Animal protocols were approved by the University of Western Ontario Animal Care Committee.

### 2.2 Serum biochemistry and liver function tests

Plasma aspartate aminotransferase (AST) and alanine aminotransferase (ALT) were quantified using a VetScan VS2 chemistry analyzer (Abaxis, Union City, CA, USA) according to the manufacturers recommendations. Plasma urea and creatinine were measured by the London Laboratory Services group using standard methods (London, ON, Canada).

Indoxyl sulfate (*m/z* 212.0018), P‐cresyl sulfate (*m/z* 187.0065), hippuric acid (*m/z* 178.0504), and phenyl sulfate (*m/z* 172.9909) were quantified using Ultra Performance Liquid Chromatography (UPLC) coupled to Quadrupole Time of Flight (QToF) mass spectrometry in accordance to the following protocol. Samples were prepared using 1:3 plasma to acetonitrile to precipitate protein, followed by centrifugation at 14 000 *g*. The supernatant was diluted 1:4 with milliQ water before injection on the Waters ACQUITY UPLC I‐Class system. Chromatography was performed on a WATERS ACQUITY UPLC HSS T3 column (100 mm × 2.1 mm, 1.8 μmol/L particle size). Water + 0.1% formic acid (A) and acetonitrile + 0.1% formic acid (B) comprised the mobile phase, which had a flow rate of 0.45 mL/min. The UPLC gradient was as follows: 0‐2 minutes 1%‐60% B; 2‐2.5 minutes 60% B; 2.5‐3.5 minutes 80% B; 3.5‐4.5 minutes 1% B. Mass spectrometry was performed using a Waters Xevo^™^ G2S‐QToF mass spectrometer in negative ESI mode with the following parameters: capillary voltage, 2 kV; cone voltage, 40 V; source temperature, 150°C; desolvation gas flow, 1000 L/h; cone gas flow, 50 L/h. Data were acquired using an MS^e^ centroid method with a 0.05 second scan time and a 50‐1200 m/z range. Collision energy was set to 15 V and ramped to 50 V. Mass accuracy was maintained using a lockspray of leucine‐enkephalin (500 ng/mL) measured every 10 seconds with a scan time of 0.3 seconds and averaged over 3 scans.

Concentration was determined by comparing sample peaks to a standard curve of indoxyl sulfate (0‐675 μmol/L), p‐cresyl sulfate (0‐250 μmol/L), hippuric acid (0‐375 μmol/L), and phenyl sulfate (0‐450 μmol/L). Quality control samples were injected every five samples and accuracy was calculated as follows: indoxyl sulfate (9.0%), p‐cresyl sulfate (4.3%), hippuric acid (11.0%), and phenyl sulfate (5.9%).

### 2.3 qPCR

Total RNA extraction was completed by using TRIzol reagents following manufacturers instructions (Life Technologies, Waltham, MA, USA). Spectrophotometry was used to assess quality and quantity of RNA. Synthesis of cDNA was performed from total RNA using qScript cDNA supermix, which contained reverse transcriptase. The qPCR reaction mix was prepared in accordance with iScript RT‐qPCR Supermix instructions (Quanta Biosciences, Inc., Gaithersburg, MD, USA). Primer sets were as follows: CYP2B1 (FW: GCTCAAGTACCCCCATGTCG, RV: ATCAGTGTATGGCATTTTACTGCGG), CYP2B2 (FW: GTACCCCCATGTCACAGAGAAA, RV: CATCAAGGGATGGTGGCCT), CYP2B6 (FW: ATGGGGCACTGAAAAAGACTGA, RV: AGAGGCGGGGACACTGAATGAC), Beta‐Actin (FW: ACGAGGCCCAGAGCAAGA, RV: TTGGTTACAATGCCGTGTTCA). The NCBI Primer BLAST program was utilized to design PCR probes. Primer sequences were designed to flank the gene of interest and were tested for specificity using gel electrophoresis and evaluation of melt curves. Primer amplification efficiency was assessed using serial dilutions of cDNA and subsequent determination of the cycle threshold at each dilution, 80% efficiency was the minimum threshold for a primer set to be used. The ΔΔCT method was used to normalize gene expression to beta‐actin.

### 2.4 Western blot

To assess expression of CYP2B1 and CYP2B2, hepatic microsomes were isolated as previously described.^45^ Twenty micrograms of protein per sample was electrophoresed on a 10% polyacrylamide gel containing 0.1% SDS. The microsomal proteins were subsequently transferred to nitrocellulose in preparation for immunoblotting. Antibodies were used according to manufacturer's specifications: The primary anti‐CYP2B antibody was diluted 1:2000 in PBS‐T with 5% skim milk (monoclonal mouse anti‐rat; purchased from Detroit R&D Inc. Detroit, MI, USA, catalogue number: P2B12PTS), while the secondary antibody (Santa Cruz Biotechnology, Dallas, TX, USA, catalogue number: SC‐51625) was diluted 1:10 000 with 5% skim milk. Horseradish peroxidase conjugation to the secondary antibody and to the beta‐actin primary antibody (monoclonal mouse anti‐beta‐actin, diluted 1:50 000 in PBST with 0.6% bovine serum albumin; Sigma Aldrich, St. Louis, MO, USA, catalogue number: A3854) allowed visualization of protein bands when Luminata Forte Western HRP substrate was applied to the nitrocellulose containing protein. Densitometry was used to quantify band intensity for determining relative changes in protein expression. Bands at 55 and 56 kDa represented CYP2B1 and CYP2B2. Due to their close proximity and difficulty with satisfactory separation, both the 55 and 56 kDa bands were combined to produce the results on CYP2B expression. A common band was found between 60 and 62 kDa but was not included in densitometry analysis.

### 2.5 Microsomal metabolism

Microsomes were aliquoted in duplicate at a concentration of 0.5 mg/mL into 96‐well plates along with microsomal incubation buffer (50 mmol/L KH2PO4 and 5 mmol/L MgCl2 at pH = 7.4). Bupropion was also added to the reaction at concentrations ranging from 10 to 500 μmol/L. NADPH at a final concentration of 1 mmol/L was added to commence the reaction, which proceeded with linear kinetics for 30 minutes. The reaction was stopped using ice‐cold acetonitrile (ACN), at a 3:1 ratio of ACN to sample. The solution contained 40 ng/mL of flurazepam to serve as an internal standard. Subsequently the samples were centrifuged at 3000 rpm for 5 minutes before being diluted 1:5 in water and subjected to mass spectrometry for quantification.

### 2.6 Hydroxy‐bupropion UPLC‐MS quantification

A standard curve was generated by serially diluting bupropion and hydroxy‐bupropion from 50 μmol/L to 50 nmol/L. Five microliters of sample was injected onto an Acquity BEH C18 column (2.1 × 50 mm, 1.7 μm) from Waters (Milford, MA, USA) maintained at 40°C. Water with 0.1% formic acid was used as solvent A, acetonitrile with 0.1% formic acid served as solvent B, and mobile phase flow was set to 0.6 mL/min. A gradient of solvent B started as isocratic at 20% until 1.75 minutes. It then increased to 50% from 1.75 to 2.5 minutes, 50%‐80% from 2.5 to 2.51 minutes, followed by 80% B for 1 minute. The lock‐mass solution was 500 pg/μL of leucine enkephalin (556.2771 *m/z* in positive ionization) in 50% acetonitrile with 0.1% formic acid, and the mass spectrometer was calibrated with 0.5 mmol/L sodium formate. The lock spray operated with a scan time of 0.3 seconds and at an interval of 10 seconds with a mass window of 0.5 Da. Electrospray ionization parameters included a capillary spray voltage of 0.5 kV; desolvation gas flow of 1000 L/h at 600°C; cone gas flow of 50 L/h with a source temperature of 150°C; sampling cone and source offset voltages at 40 and 80 V, respectively. Data were acquired using positive ionization and the instrument was run in resolution mode with a mass range from 50 to 1200 Da with 0.1 scans per second.

### 2.7 Data and statistical analysis

Statistical analysis was completed by unpaired, two‐tailed *t* tests to compare control and CKD groups. Data are displayed as mean ± standard error of the mean. The threshold for significance was *P *<* *0.05 except where several metabolites were tested simultaneously in which case Bonferroni correction was applied. Statistics and graphs were generated using GraphPad Prism version 6.0 software.^25^ In regard to the microsomal metabolism assays, results were plotted using Michaelis‐Menten enzyme kinetics to calculate the metabolic parameters *K*_m_ (the Michaelis constant) and *V*_max_ (maximum velocity).

## 3 RESULTS

**Figure 1:**
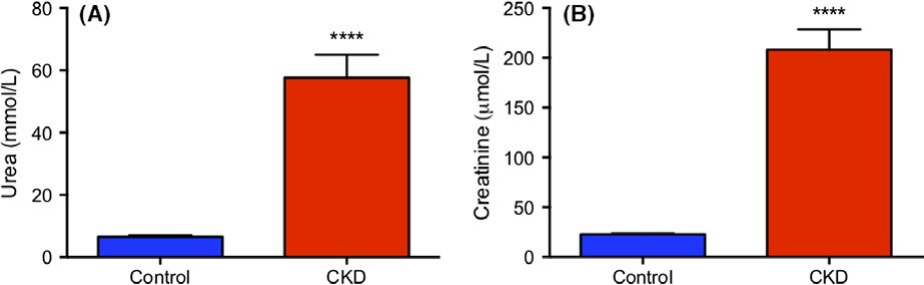
Plasma urea (A) and creatinine (B) levels in control and CKD Wistar rats, measured in mmol/L and μmol/L, respectively. Rats were fed standard chow (control) or standard chow with 0.7% adenine (CKD) for 42 days. Results are presented as mean ± SEM, n = 10‐12 per group and the threshold for significance after Bonferroni correction was 0.025, ****P <* *0.0001 compared to control

Kidney function was assessed at the end of the study by quantification of plasma urea and creatinine (Figure 1). Both plasma urea and creatinine were significantly elevated in the CKD group compared to control (*P *<* *0.0001), evident by an 8.7 and 9.1‐fold (57.7 ± 7.4 and 6.6 ± 0.5 mmol/L; 208.2 ± 20.4and 22.8 ± 0.6 μmol/L) increase in concentration, respectively.

**Figure 2:**
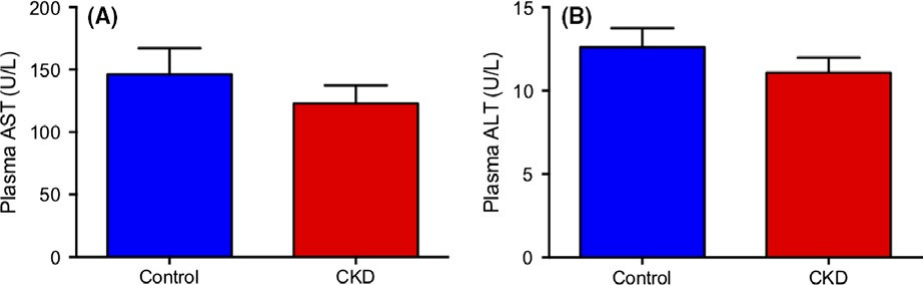
Plasma AST (A) and ALT (B) levels in control and CKD Wistar rats, measured in U/L. Rats were fed standard chow (control) or standard chow with 0.7% adenine (CKD) for 42 days. Results are presented as mean ± SEM, n = 10‐12 per group and the threshold for significance after Bonferroni correction was 0.025 (*P *=* *0.354 and *P *=* *0.308, respectively)

To ensure that the adenine diet did not produce overt liver toxicity, liver function was assessed by determination of plasma levels of the hepatic enzymes AST and ALT (Figure 2). No significant difference was seen between control and CKD groups for either AST or ALT (*P *=* *0.354 and *P *=* *0.308, respectively).

**Figure 3:**
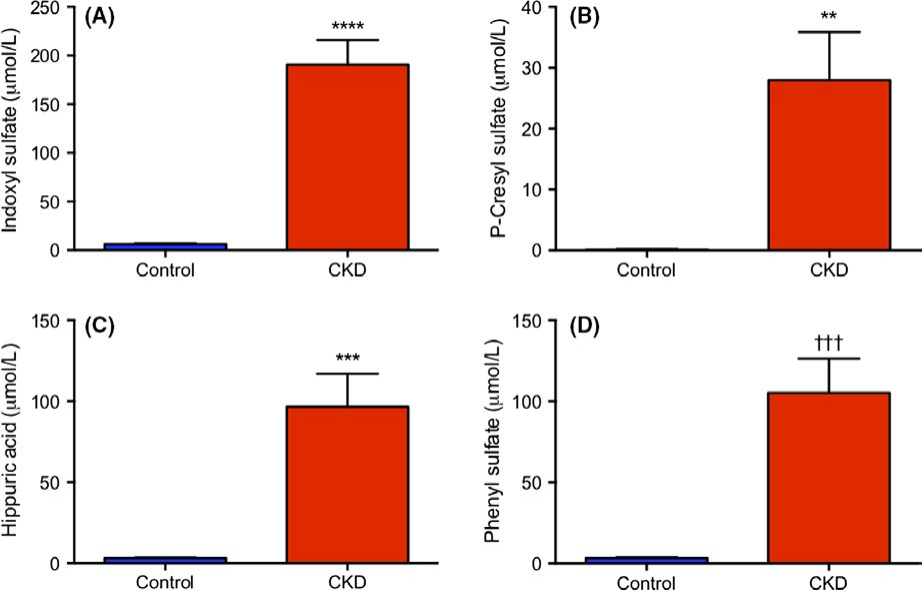
Concentrations of plasma indoxyl sulfate (A), P‐cresyl sulfate (B), hippuric acid (C), and phenyl sulfate (D) in μmol/L. Rats were fed standard chow (control) or standard chow with 0.7% adenine (CKD) for 42 days. Results are presented as mean ± SEM, n = 10‐12 per group and the threshold for significance after Bonferroni correction was 0.0125 **P =* *0.0045, ***P =* *0.0005, ^†††^ *P *=* *0.0003, and ****P <* *0.0001 compared to control

Select plasma uremic toxins were quantified to supplement model validation (Figure 3). Indoxyl sulfate was 30.6‐fold greater in the CKD group compared to control (190.8 ± 25.1 and 6.2 ± 0.8 μmol/L, respectively, *P *<* *0.0001). P‐cresyl sulfate was 232.5‐fold greater in the CKD group compared to control (27.9 ± 7.9 and 0.1 ± 0.1 μmol/L, respectively, *P *<* *0.01). Hippuric acid was 30.7‐fold greater in CKD compared to control (96.5 ± 20.4 and 3.1 ± 0.4 μmol/L, respectively, *P *<* *0.001). Phenyl sulfate was 32.3‐fold greater in CKD compared to control (105.2 ± 21.1 and 3.3 ± 0.5 μmol/L, respectively, *P *<* *0.001).

**Figure 4:**
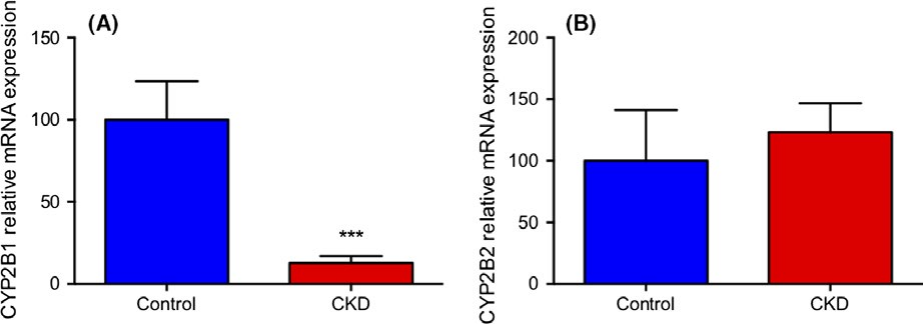
Relative hepatic mRNA expression of CYP2B1 (A) and CYP2B2 (B) in an adenine diet CKD model. The sample mRNA was extracted from total liver homogenate. Results are presented as mean ± SEM, n = 9‐12 per group, and the threshold for significance after Bonferroni correction was 0.025 ***P <* *0.001 compared to control. β‐actin was used as the housekeeping gene for CYP2B1 and CYP2B2

CYP2B1 and CYP2B2 mRNA levels from CKD and control groups were quantified using qPCR (Figure 4). Levels of CYP2B1 mRNA were markedly lower in the CKD group compared to control, accounting for an 87% decline in expression (*P *<* *0.001). No statistically significant differences in expression were seen for CYP2B2 between groups (*P *=* *0.608).

**Figure 5:**
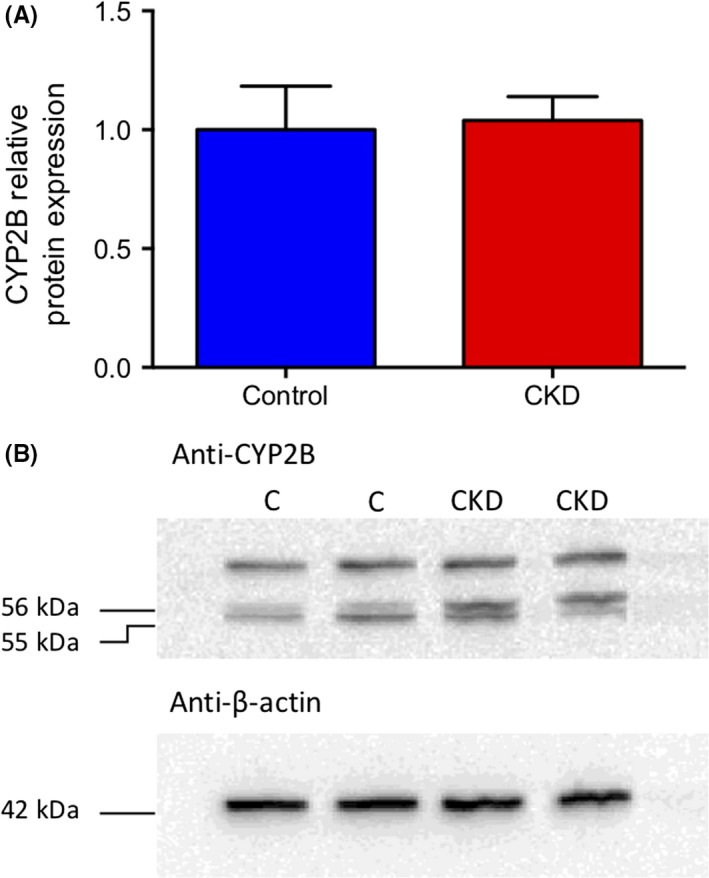
(A) Relative protein expression of CYP2B in hepatic microsomes from an adenine model of CKD in Wistar rats. Control and CKD groups are presented with mean ± SEM, n = 9‐12 per group. β‐actin was used as the housekeeping protein. (B) Representative blots with C = Control, CKD = CKD

Protein expression of CYP2B showed no significant differences between control and CKD groups (Figure 5, *P* = 0.847).

**Figure 6:**
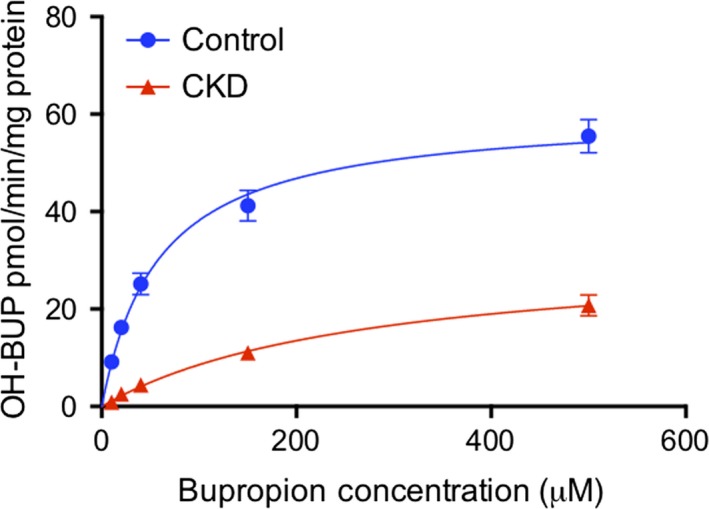
Bupropion metabolism to OH‐bupropion as a measure of CYP2B activity in rat liver microsomes from control and CKD animals (pmol/min/mg protein). *V*_max_ and *K*_m_ were determined using the Michaelis‐Menten model, n = 9‐12 per group

The rate of metabolism of bupropion by rat liver microsomes is shown in Figure 6. Rat liver microsomes from the CKD group showed significantly decreased metabolism of bupropion compared to control (*P *<* *0.05). Michaelis‐Menten analysis showed dramatically higher *K*_m_ (5.2 fold) as well as significantly lower *V*_max_ (46% decline) and intrinsic clearance (88% decline) in the CKD group relative to control (*P *<* *0.0001) (Table 1).

**Table 1:** Michaelis‐Menten kinetic parameters from control and CKD rat liver microsomes incubated with bupropion

	Control	CKD
*V*_max_ (pmol/min/mg protein)	60.7 ± 3.7	32.7 ± 2.7****
*K*_m_ (μmol/L)	60.3 ± 2.6	312.2 ± 30.3****
Intrinsic Clearance (μl/min/mg protein)	1.04 ± 0.10	0.12 ± 0.02****

Note

Presented as mean ± SEM, n = 9‐12 per group ****P <* *0.0001 compared to control.

## 4 DISCUSSION

To our knowledge, the expression and function of CYP2B1 and CYP2B2 has not been previously evaluated in the setting of CKD. Although CYP2B1 and CYP2B2 are rat‐specific enzymes, there is a high sequence homology with human CYP2B6.^22^ The importance of CYP2B6 in human drug metabolism is emphasized in the literature due to its contribution to the biotransformation of drugs such as cyclophosphamide, bupropion, clopidogrel, and efavirenz^2^, ^13^, ^36^; H‐J.^3^, ^10^, ^22^, ^50^

The in vivo model of CKD was developed using a 0.7% adenine diet in male Wistar rats. Measurement of plasma urea and creatinine were 8.7 and 9.1‐fold higher in the CKD group, respectively, which validated the presence of renal damage in the model. In addition, plasma concentration of the uremic toxins indoxyl sulfate, p‐cresyl sulfate, hippuric acid, and phenyl sulfate were also significantly elevated in the CKD group. This difference in plasma uremic toxins supports that the 0.7% adenine supplemented rats had decreased renal function and that their plasma resembled the uremic milieu seen in CKD patients. Previous model validation and results from the current study reported no increase in liver function enzymes AST or ALT in CKD animals compared to control.^8^, ^46^ Accordingly, the observed changes in liver CYP2B1 expression and function are not attributed to direct hepatic damage. A significant change in mRNA level of CYP2B1 was observed as mRNA levels in CKD animals were decreased by 87.3% relative to control. The mRNA level of CYP2B2 was unchanged between CKD and control. Follow‐up experiments to assess protein expression of CYP2B showed no difference between disease and control. This result may be due to nonspecific antibody binding, since CYP2B1 and CYP2B2 share 97% sequence homology. Based on this, the unchanged CYP2B protein level between control and CKD may not accurately depict CYP2B1 expression, which was unable to be determined by Western blot. To determine the effect of CKD on CYP2B1 protein level, future studies using mass spectrometry to specifically quantify CYP2B1 concentration are required.

Although overall CYP2B protein expression appeared unchanged, the pronounced decrease in mRNA expression spurred further exploration into the metabolic activity of CYP2B1 as it is noted to be more catalytically active than CYP2B2.^31^ BUP was selected as the probe substrate for CYP2B1 due to previous work validating its high metabolism by this enzyme.^31^ A significant decrease was observed in the function of CYP2B1 in CKD compared to control, which was exemplified by reductions in *V*_max_ and intrinsic clearance by 46.2% and 88.4%, respectively.

The decrease of BUP conversion to OH‐BUP in CKD rats supports the mRNA data since CYP2B1 is the primary metabolic enzyme for this compound.^31^ The significant decreases in *V*_max_ and intrinsic clearance can be explained by the loss of CYP2B1 expression. The marked increase in *K*_m_ is likely reflective of the activity of other CYP isoenzymes that have lower affinity for bupropion (eg, CYP2C11 or CYP2E1). CYP2B1 normally accounts for 75% of OH‐BUP production while CYP2C11 will metabolize approximately 11% of BUP.^31^ In our model, CYP2B1 expression is decreased and the activity of CYP2C11 has been shown to decrease significantly in CKD as well.^19^, ^45^ A study performed on rat primary hepatocytes which were incubated with serum from CKD patients showed no difference in CYP2E1 expression, which supports this theory.^24^ Therefore, we suggest that the production of OH‐BUP in microsomes from rats with CKD may be mediated by CYP isoforms, such as CYP2E1 that are unaffected by CKD and have a low affinity for BUP.

The clinical significance of altered CYP2B1 expression and function lies with its homology to human CYP2B6. A study published in 2007 assessed BUP conversion to OH‐BUP in patients with renal impairment. They were unable to show a direct effect of CKD on BUP metabolism but did record increases in half‐life and area under the plasma‐concentration time curve (AUC) (140% and 126%, respectively) corresponding with an apparent reduction in clearance of 63% in CKD patients.^42^ Based on experimental conditions the evidence suggested that CYP2B6 activity was decreased, but the authors could not unequivocally state this claim. A second study in 2010 demonstrated decreased BUP and OH‐BUP clearance in patients with glomerulonephritis, insinuating that patients with glomerular disease also have decreased CYP2B6 activity.^14^ Furthermore, the pharmacokinetics of BUP were similar in a study that compared BUP metabolism in hemodialysis patients who smoked to a control population with normal renal function.^49^ However, there was a much larger AUC for OH‐BUP in the end‐stage renal disease (ESRD) group compared to control, indicating there was accumulation of the drug in these patients.^49^ An in vitro study in 2014 showed no change in human microsomal CYP2B6 function when incubated with uremic serum from human ESRD patients pre‐ and postdialysis.^47^ The authors did note some limitations of the study which included a small sample size (n = 10) and an entirely African‐American subject base despite no case‐control matching incorporated into the study design. Patients from that study also received dialysis three times per week, which may have nullified an inhibitory effect of uremic serum on metabolism due to insufficient time allowed for toxin accumulation. The authors also suggested that their use of ultrafiltered serum may have removed some of the more strongly protein‐bound uremic toxins which have been shown to have interactions with CYP enzymes in other studies.^11^, ^41^, ^47^ The study by Volpe et al from 2014 also supports the notion that altered CYP2B function is unlikely to be due to direct inhibition from uremic toxins.

In conclusion, clinical pharmacokinetic studies in patients with CKD have demonstrated altered bupropion disposition, which can be attributed to altered CYP2B6 activity. Our findings demonstrate that CYP2B1 mRNA expression is significantly decreased in a rat model of CKD. In addition, our rat model of CKD caused a decrease in bupropion metabolism. Our data suggests that the altered bupropion disposition observed in human patients with CKD is mediated by decreased CYP2B expression. In addition, our data supports the findings from several other studies that show a decreased expression of P450 enzymes in kidney disease. It is likely that uremic toxins retained in kidney disease mediate the observed downregulation of expression. Future studies should determine if there are common mechanisms of P450 downregulation between enzymes such as CYP2B1 described in this study with CYP2C11 and CYP3A2, which have been consistently shown to be downregulated in similar models of CKD. Understanding which uremic mediators cause this downregulation may help develop strategies to normalize hepatic P450 expression in kidney disease as these enzymes are important for not only drug metabolism, but also for hormone and steroid metabolism. Alternatively, studies directed toward altered drug dosing for substrates of CYP2B6 can help to lower the occurrence of subtherapeutic or toxic dosing events in patients with CKD.

## DISCLOSURES

None declared.

## AUTHOR CONTRIBUTIONS

Kucey, Velenosi, and Urquhart participated in research design. Kucey, Velenosi, Tonial, Tieu, and RaoPeters conducted experiments. Urquhart contributed new reagents or analytical tools. Kucey, Velenosi, and Tonial performed data analysis. Kucey, Velenosi, Tonial, Tieu, and Urquhart wrote or contributed to the writing of the manuscript.
